# A New Automated Method and Sample Data Flow for Analysis of Volatile Nitrosamines in Human Urine^*^

**DOI:** 10.4236/ajac.2016.72014

**Published:** 2016-02-02

**Authors:** James A. Hodgson, Tiffany H. Seyler, Ernest McGahee, Stephen Arnstein, Lanqing Wang

**Affiliations:** 1 Tobacco and Volatiles Branch, Division of Laboratory Sciences, National Center for Environmental Health, Centers for Disease Control and Prevention, Atlanta, USA; 2 Oak Ridge Institute for Science and Education (ORISE), Oak Ridge, USA

**Keywords:** Volatile Nitrosamines, Automation, Sample Data Flow, Gas Chromatography, Tandem Mass Spectrometry

## Abstract

Volatile nitrosamines (VNAs) are a group of compounds classified as probable (group 2A) and possible (group 2B) carcinogens in humans. Along with certain foods and contaminated drinking water, VNAs are detected at high levels in tobacco products and in both mainstream and sidestream smoke. Our laboratory monitors six urinary VNAs—N-nitrosodimethylamine (NDMA), N-nitrosomethylethylamine (NMEA), N-nitrosodiethylamine (NDEA), N-nitrosopiperidine (NPIP), N-nitrosopyrrolidine (NPYR), and N-nitrosomorpholine (NMOR)—using isotope dilution GC-MS/MS (QQQ) for large population studies such as the National Health and Nutrition Examination Survey (NHANES). In this paper, we report for the first time a new automated sample preparation method to more efficiently quantitate these VNAs. Automation is done using Hamilton STAR^™^ and Caliper Staccato^™^ workstations. This new automated method reduces sample preparation time from 4 hours to 2.5 hours while maintaining precision (inter-run CV < 10%) and accuracy (85% - 111%). More importantly this method increases sample throughput while maintaining a low limit of detection (<10 pg/mL) for all analytes. A streamlined sample data flow was created in parallel to the automated method, in which samples can be tracked from receiving to final LIMs output with minimal human intervention, further minimizing human error in the sample preparation process. This new automated method and the sample data flow are currently applied in bio-monitoring of VNAs in the US non-institutionalized population NHANES 2013-2014 cycle.

## 1. Introduction

**Figure 1:**
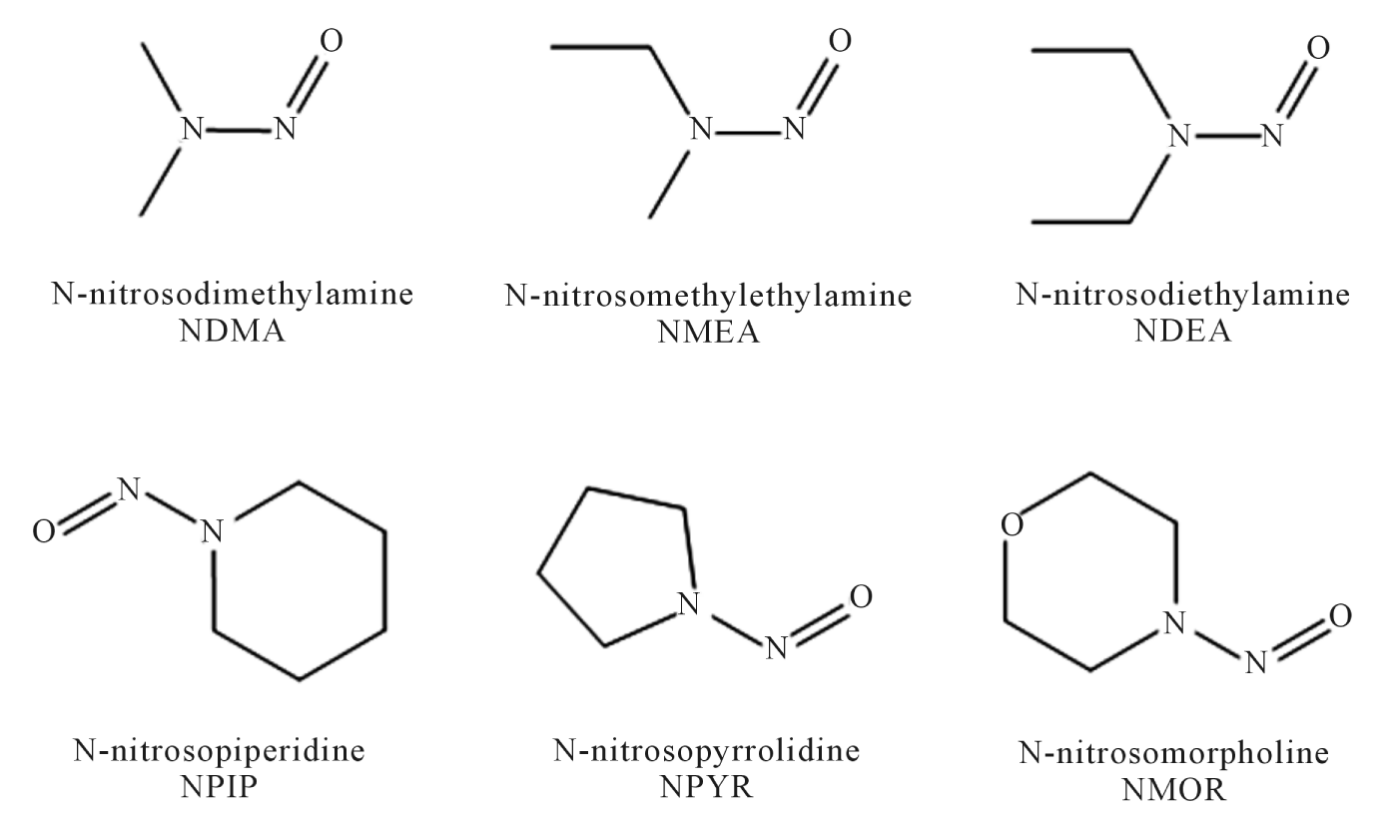
Structures of volatile nitrosamines.

Volatile nitrosamines (VNAs) are a class of nitrosated secondary and tertiary amines (Figure 1). VNAs are known carcinogens and teratogens in animals and are classified as group 2A and 2B carcinogens in humans [1]-[9]. They have been shown to induce tumors via cytochrome-activated DNA alkylation in several organs, including liver, lungs, kidney, bladder, pancreas, and esophagus [1] [5]-[7] [9] [10]. VNAs may lead to lipid peroxidation and oxidative stress, as well as chronic diseases such as diabetes and Alzheimer’s disease [11]-[18].

The formation of volatile nitrosamines occurs through the nitrosation of secondary and tertiary amines via interaction with nitrite, which itself is a product of nitrate reduction [3]-[5] [7] [8] [19] [20]. For this reason, VNAs can be formed from many items containing nitrates and nitrites, such as cured meats, fish products, cosmetics, certain types of beers, and tobacco products (as part of the curing process and during product assembly), as well as in both mainstream and sidestream tobacco smoke [2] [4] [7] [21]-[23]. Another significant source of VNA exposure can be drinking water: VNAs, particularly NDMA, can form as byproducts during disinfection via chlorination and chloramination [2] [9] [24]-[28].

Many different methods are reported for VNA measurements. For sample preparation, dichloromethane is the solvent of choice for extracting VNAs from the sample matrix (e.g. water, urine, serum), whether in a direct liquid-liquid extraction or using a solid phase intermediary [9] [24]. As for instrumentation, gas chromatography is the most common separation technique due to the eponymous volatility of VNAs, though some labs have developed various liquid chromatography methods [2] [5] [6] [22] [23] [27]. Detection methods range from single- and triple-quadrupole mass spectrometry to thermal energy analysis and nitrogen chemiluminescence detection, though mass spectrometry is the most commonly used [1] [8] [19] [20] [25] [26] [28].

Automation is necessary for higher sample throughput in large population studies such as NHANES, whose sample size is approximately 10,000 per two-year cycle. In this study, we present an automated method utilizing both Caliper Staccato and Hamilton Star workstations. The throughput is increased by automation equipment, and the sensitivity is increased due in part to an upgrade of the QQQ from an Agilent 7000B to a 7000C. We created a streamlined sample data flow in parallel to the automated method, in which samples can be tracked from receiving to final LIMs output with minimal human intervention, further minimizing human error in the sample preparation process.

## 2. Methods and Materials

### 2.1. Materials

Native standards were purchased from Supelco (Sigma-Aldrich, St. Louis, MO) as a 2 mg/mL mixture in dichloromethane (DCM). Deuterium-labeled internal standards NDMA-D6, NDEA-D10, NPYR-D8, and NMOR-D8 were purchased individually in DCM from Cambridge Isotope Laboratories (Tewksbury, MA); NMEA-D3 and NPIP-D10 were purchased individually as pure oils from Toronto Research Chemicals (Toronto, Canada). Methanol (MeOH), DCM, and acetonitrile (ACN) were HPLC grade, purchased from Honeywell Burdick & Jackson (Muskegon, MI). Sample plates were Axygen 48-well plates with a 5 mL well capacity. GC vials were Wheaton 11 mm amber crimp vials with a 300 μL insert; crimp caps were SUN-SRi 11 mm aluminum crimp caps with rubber septum. All GC-QQQ parts were purchased from Agilent Technologies (Santa Clara, CA).

### 2.2. Hamilton Microlab Star Liquid Handling Workstation

All sample and internal standard aliquoting was performed on the Hamilton Microlab Star liquid handling workstation. The pipetting array consisted of 8 pipetting heads and used 1000 μL and 50 μL compressed O-ring expansion (CO-RE) tips. All samples were aspirated using capacitance liquid level detection (cLLD) and dispensed using the jet empty setting. Samples were mixed (consisting of an aspirate of 500 μL and dispense into the same container) 3 times prior to being transferred to the sample plate to ensure sample uniformity.

### 2.3. Caliper Staccato Specimen Handling Workstation

**Figure 2:**
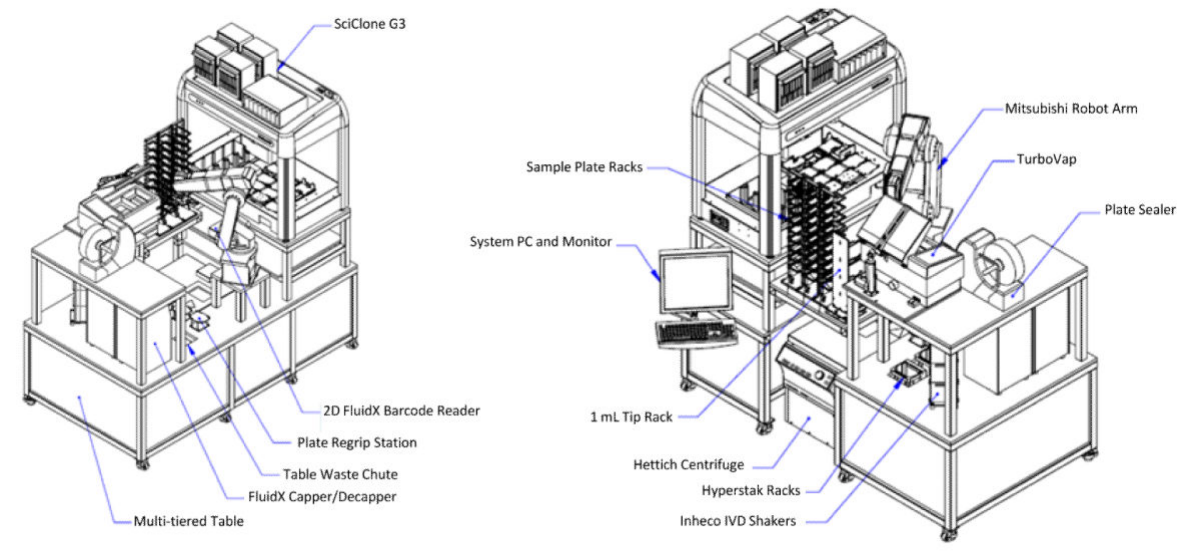
Schematic for Caliper Staccato workstation.

Sample preparation was performed on the Caliper Staccato specimen handling workstation, including an integrated Perkin Elmer SciClone G3 Automated Liquid Handling Workstation (Figure 2). This system was custom designed with all specimen preparation needs in mind. The Staccato station includes the SciClone G3 Workstation, a Biotage TurboVap 96 Automated Evaporation System, a Hettich GmbH & Co. KG Rotanta 460 Robotic Centrifuge, two HyperStak39 consumable loaders, a Thermo Scientific ALPS 3000 sealer, and a Mitsubishi S Series Melfa RV-6SDL Industrial Robot.

### 2.4. Automated Sample Preparation

Urine samples were transferred from cryovials in 2 mL aliquots, along with 500 pg of internal standard (in methanol solution, 10 pg/μL), into a 48-well plate using a Hamilton Star liquid handling system. The delivered volume of 50 μL was verified gravimetrically with % error of less than 1%. The sample plate was transferred to a SciClone liquid handler on the Caliper Staccato specimen handling workstation, where 2.5 mL of DCM were added to each well. All samples underwent pipette mixing for approximately 40 minutes using the 96-head main array and 200 μL pipette tips. The plate was sealed using a Thermo Scientific ALPS 3000 sealer and centrifuged for 5 minutes (1500 rpm, 25°C) in a Hettich Rotanta 460 Robotic centrifuge. The sample plate was then moved back onto the SciClone deck, where the seal was pierced, and 1.5 mL of the DCM layer in each well were transferred to a new 48-well plate. Samples were evaporated to approximately 300 μL in a Biotage 96-array TurboVap, and the plate was sealed and transferred off the Caliper Staccato system. Each sample was then manually transferred via pipette to a 1 mL amber GC vial with a 300 μL insert and further evaporated to approximately 100 μL in a ThermoFisher Savant SPD2010 Speedvac Concentrator. To each sample 50 μL of ACN were added, then the remaining DCM was evaporated in the Savant. Sample vials were sealed using aluminum crimp caps and transferred to GC-MS/MS (QQQ) for analysis (Figure S1).

### 2.5. GC-MS/MS (QQQ) Analysis

All analyses were performed on an Agilent 7890-7000C GC-MS/MS (QQQ). The 7890 GC was equipped with a multimode inlet (MMI) and a single taper helix liner. The injection volume was 5 μL. The initial injection temperature was 5°C, which was held for 0.85 minutes after injection and then heated at 600°C/min to 300°C. A programmed temperature vaporation (PTV) solvent vent mode was used, venting at 200 mL/min flow rate and 5 psi pressure for 0.7 minutes after injection. A two-column setup connected by a backflush union was used for the GC, with helium as the carrier gas. The first column was an Agilent DB-WAXetr (30 m × 0.25 mm × 0.5 μm) with a constant flow rate of 3 mL/min for the first 1.3 minutes, followed by a 1.2 mL/min flow rate for the remainder of the run. The second column was deactivated fused silica (1 m × 0.15 μm) with a constant pressure of 1 psi. The GC oven was initially set to 35°C for 1 minute after injection then heated to 245°C at 20°C/min. A backflush was performed for 5 minutes post-run, with a −1.9795 mL/min constant flow for the first column, a 25 psi constant pressure for the second column, and an oven temperature of 250°C. The transfer line temperature and MS source were both set to 250°C. The 7000C MS source mode was positive chemical ionization (CI) with ammonia (blue grade) as the CI gas; ultra-high purity nitrogen was used as the collision gas.

### 2.6. Sample Data Flow

**Figure 3:**
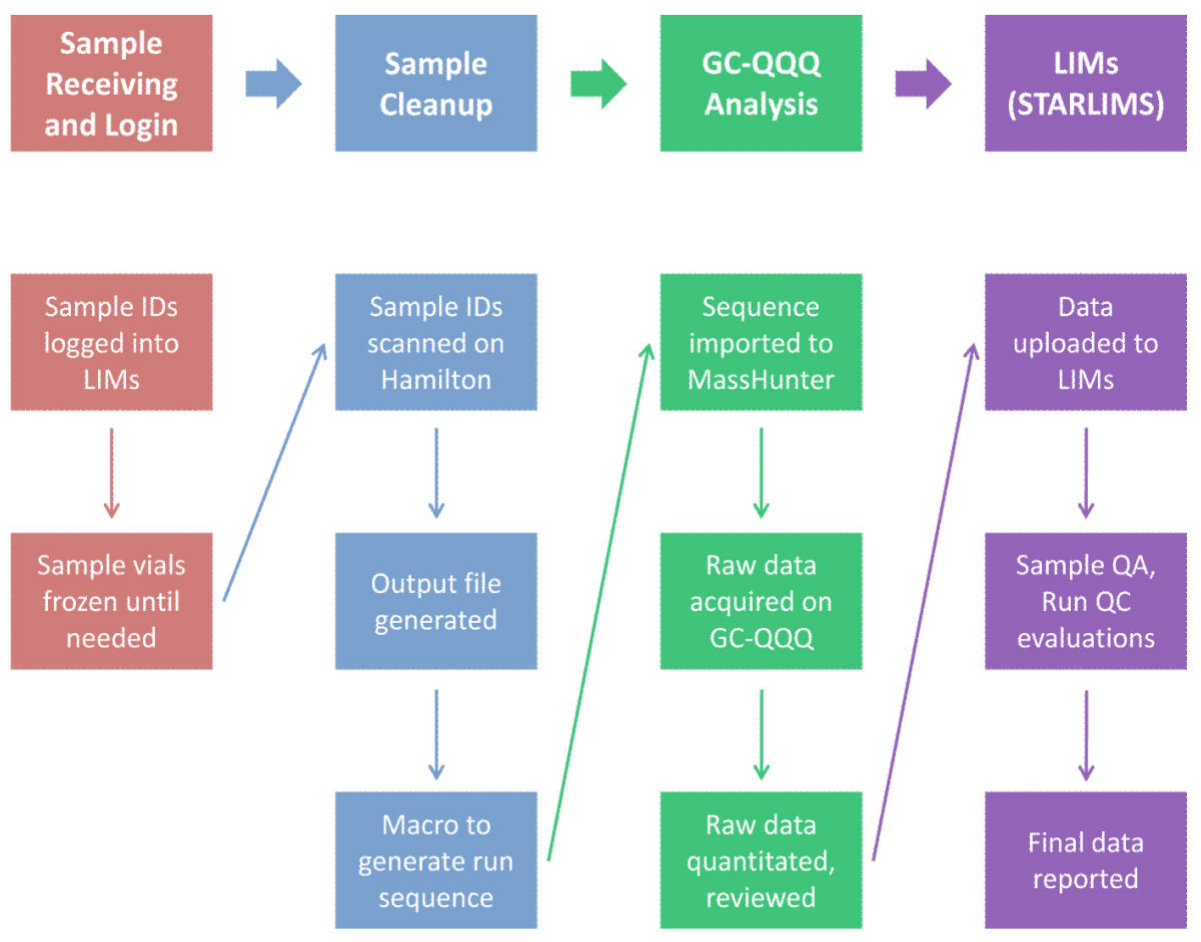
Sample data flow chart.

A mostly automated system for tracking sample data was created in parallel with the automated sample preparation method (Figure 3). Samples are received and logged into the LIMs reporting system before they are queued for preparation and analysis. Samples to be prepared are scanned by the Hamilton Star, which upon completion generates an output file mapping scanned samples to positions in the 48-well plate. This Hamilton output file is run through an Excel macro which modifies the format so that it can be imported into MassHunter as a sequence file. Once the raw data are acquired from the GC-QQQ, they can either be analyzed in MassHunter Quantitative software or uploaded to Indigo Biosystems ASCENT platform for automatic integration. Regardless of which quantitation software is used, a formatted output file containing the final calculated data is generated and can be directly uploaded to the LIMs system. Using a custom LIMs system, unknown samples are evaluated individually according to a list of QA rules, including retention times of internal standard and main ion transition peaks, confirmation ion ratio, internal standard peak area, and blank limit. Batch QCs are evaluated according to modified Westgard QC rules [29]. Final results are then exported to a final reporting system such as NHANES.

## 3. Results

### 3.1. Blanks

A true blank has proven difficult to produce, as VNAs—particularly NDMA—are detected in all water sources tested thus far, including Fluka Analytical *Trace*SELECT ultratrace water (Sigma-Aldrich), a commercial source of VOC-free water, and in-house filtered “VOC-free” water. NDMA contamination has also been detected when DCM (the extraction solvent) comes into contact with any plastic consumable used during the sample preparation process, such as the 48-well plates and pipette tips. As a result, DCM is processed as an unknown sample and used as the system blank and for blank subtraction. The concentration of NDMA contamination seen in DCM blank samples is approximately 1 - 2 times the LOD reported here. The first and last well in the plate contain DCM, spiked with 500 pg ISTD, and carried all the way through the sample preparation process. The average calculated concentration derived from the analytical results of these two DCM blanks is then subtracted from all analytical results for the remainder of that batch (i.e. all other samples prepared in the same 48-well plate). The blank subtraction is built into the process of data loading to LIMs. Blank characterization was established over 60 separate runs over a one year period. Runs are rejected if any DCM blank result exceeds the established blank limits for each analyte.

### 3.2. Carryover

Acetonitrile blanks were run immediately after high (200 pg/mL) QC samples. These blanks were then compared with blanks run without any immediately preceding sample. Calculated concentrations of the two sets of blanks were within 5% both of one another and of the blank characterization values, indicating no carryover. Some small carryover was observed for NDMA, NMEA, and NDEA after the injections of the highest standard, 400 ng/mL. After 3 ACN solvent blank injections, there is no carryover observed for these three analytes. Thus, ACN solvent blank is injected three times after the highest standard in every analytical batch. As a precaution, a QA rule is built into the LIMs system to flag a sample immediately following a high concentration sample (>200 pg/mL). The flagged samples are reinjected to ensure no carryover occurred. Carryover is determined by established repeatability criteria: 20% for lower concentration (<50 pg/mL) and 10% for higher concentration (≥50 pg/mL). If a reinjected sample fails repeatability rules, it will be repeated. An ACN blank is also injected at the beginning of each analytical batch to ensure no system contamination exists prior to sample analysis.

### 3.3. Limits of Detection (LOD)

The limit of detection for each analyte was obtained from 60 independent runs (limited to only 1 run per day) using DCM blanks (carried through sample preparation process, as mentioned above), as well as prepared samples from 4 pools: 0 pg/mL, 2.5 pg/mL, 5.0 pg/mL, and 7.5 pg/mL. Because NDMA, NMEA, NDEA, NPIP, and NMOR have positive blank detections, these LODs were calculated using the 3S_0_ method, where S_0_ is the extrapolated standard deviation at zero concentration. For NPYR, the LOD was determined according to the guideline for determination of limits of detection by the Clinical and Laboratory Standard Institute using the 4 QC pools [29]. The LODs for all analytes are below 10 pg/mL, with three of the analytes (NMEA, NDEA, and NPIP) at or below 5 pg/mL (Table 1).

### 3.4. Precision

To determine intra-run and inter-run precision, in-house anonymous non-smoker urine (collected with CDC Institutional Review Board (IRB) approval) was spiked to make two QC pools at 50 pg/mL and 200 pg/mL. Six samples of each QC were run for five consecutive days. For the 50 pg/mL QC, only one analyte in one run has an intra-run CV greater than 10%; the rest of the pool is below 7% CV. For the 200 pg/mL QC, all analytes are at or below 5% intra-run CV. For both sets, all inter-run CVs are 5-10% (Table 2).

### 3.5. Accuracy in Solution

To determine accuracy of the calibration curve, individual stocks of native NDMA, NDEA, NPYR, and NMOR were obtained from a different vendor, Cambridge Isotope Laboratories. Because no manufacturer readily carries individual stocks of native NMEA or NPIP, a different lot of the Supelco native VNA mixture was obtained. Three levels of calibrator (at 0.5%, 25%, and 50% of the highest calibrator) were made and run in triplicate for each individual stock, as well as for the new mixture. For all three levels, the accuracy is greater than 94% for all analytes, both individually and in the mixture (Table 3). This test is repeated every time a new standard curve is prepared for analysis.

### 3.6. Accuracy in Matrix

To determine accuracy in matrix, freshly collected in-house anonymous non-smoker urine (collected with CDC Institutional Review Board (IRB) approval) was spiked at 3 different levels each day: 100 pg/mL, 200 pg/mL, and 300 pg/mL. These pools were prepared and run in triplicate for three consecutive days. For the 100 pg/mL spiked samples, the calculated accuracy for all analytes is 85% - 111%; for 200 pg/mL and 300 pg/mL, the accuracy for all analytes is 92% - 106% (Table 4).

## 4. Discussion

Automation of sample preparation processes is a crucial part of bio-monitoring large population studies like NHANES. Analyzing more than 10,000 samples per two-year cycle requires a much higher throughput than an analyst could perform manually. Our new automated method for preparing samples enables the analyst to achieve the necessary sample throughput while still maintaining high accuracy and precision. With detection limits for all analytes below 10 pg/mL, and three of six at or below 5 pg/mL, these LODs are comparable to the ones reported in Seyler, *et al*. [21]. Also, the staff time saved because of the automated steps can be re-allocated to instrument operation and data analysis, further increasing throughput.

The automated sample data flow further minimizes human error in sample handling. Sample IDs are first tracked during initial sample aliquoting on the Hamilton Star, where an output file automatically maps samples to well locations on the 48-well plate. The Hamilton output file is then converted to an imported GC-QQQ sequence file via an Excel macro. All relevant sample information, such as the sample ID, sample volume, and any dilution factors, is saved with each raw data file as the samples are analyzed on the GC-QQQ. The current method allows for one of two data analysis processes to occur. Currently, all sample data is analyzed using MassHunter Quantitative Analysis software. The ability to manually integrate peaks from baseline to baseline makes this method the most accurate for samples at low levels, especially around the LODs. The second data analysis process, Indigo Biosystems ASCENT platform, is a more automated process but is still being optimized. The peak fitting and peak picking algorithms utilized on this platform work well for higher concentration samples. The one main impediment to using this platform thus far is the inability to adjust peak baselines; the software only allows for two data points to be chosen on the chromatogram, and the area between is automatically integrated. This has been of greatest concern in samples around the LODs of all analytes: the algorithm for determining the chromatogram baseline creates an undulating baseline, which when picking a small peak can artificially increase or decrease the calculated concentration. Indigo is currently addressing this problem for this method, and if it can be resolved, then the Indigo ASCENT platform will be implemented in the main data analysis process.

## 5. Conclusion

Overall, the newly automated process is time efficient and precise. At least two batches of 48 samples can be prepared each day when higher sample throughput is needed. Automation has improved the overall accuracy and precision. The new sample data flow has improved sample tracking and data analysis, including sample and run quality control evaluation. The sample data flow also enables multiple team members to participate and track sample analysis progress. The entire sample data flow from sample receiving to final result reporting is more efficient while minimizing human errors. This method will be implemented to monitor volatile nitrosamines in population studies such as NHANES.

## Supplementary Material

01 **Figure S1.** Sample preparation work flow.
**Figure S2.** Short-term stability of NDMA.
**Figure S3.** Short-term stability of NMEA.
**Figure S4.** Short-term stability of NDEA.
**Figure S5.** Short-term stability of NPIP.
**Figure S6.** Short-term stability of NPYR.
**Figure S7.** Short-term stability of NMOR.
**Table S1.** Matrix equivalency (All r^2^ values > 0.999).
**Table S2.** Ruggedness.

## Figures and Tables

**Table 1:** Limits of detection of all analytes (LODs were obtained from 60 independent runs, limited to one run per day).

	Limit of Detection (pg/mL)
NDMA	6.54*
NMEA	3.64*
NDEA	5.02*
NPIP	5.08*
NPYR	8.15**
NMOR	7.84*

* Determined by 3S_0_, where S_0_ is the standard deviation of blank characterization;

** Calculated according to CLSI, using 4 pools (0 pg/mL, 2.5 pg/mL, 5.0 pg/mL, 7.5 pg/mL).

**Table 2:** Precision.

Pool 1—50 pg/mL						

	NDMA	NMEA	NDEA	NPIP	NPYR	NMOR
Intra-run (n = 6) CV (%)	4.26	4.87	4.63	1.90	4.65	4.69
	5.26	2.03	3.60	4.51	6.43	2.43
	3.77	2.42	2.68	1.05	6.54	2.18
	2.03	2.68	1.93	2.19	3.19	3.16
	11.1	3.02	5.16	5.04	5.44	2.54
Inter-run (n = 5)	7.94	6.14	5.17	5.36	9.34	5.16

Pool 2—200 pg/mL						

	NDMA	NMEA	NDEA	NPIP	NPYR	NMOR
Intra-run (n = 6) CV (%)	3.40	2.24	2.68	1.97	2.68	5.07
	1.58	1.26	1.69	2.19	2.08	1.24
	1.69	2.91	2.33	2.67	2.75	2.49
	1.49	2.16	2.76	2.98	2.29	2.20
	3.97	3.27	3.55	3.23	4.16	3.32
Inter-run (n = 5)	7.44	7.09	6.78	6.95	5.70	6.12

**Table 3:** Accuracy in solution.

Accuracy (%) n = 3	NDMA	NMEA	NDEA	NPIP	NPYR	NMOR
Mixed—2 ng/mL	99.9	95.5	95.4	94.8	96.2	94.2
Mixed—100 ng/mL	100	101	100	101	98.7	102
Mixed—200 ng/mL	101	103	101	100	98.7	102
Individual—2 ng/mL	95.7		94.7		96.7	98.0
Individual—100 ng/mL	94.3		99.1		96.8	104
Individual—200 ng/mL	96.7		101		98.8	104

**Table 4:** Accuracy in matrix.

100 pg/mL			200 pg/mL			300 pg/mL		

n = 9	AVG (%)	CV (%)	n = 9	AVG (%)	CV (%)	n = 9	AVG (%)	CV (%)
NDMA	85.4	2.56	NDMA	94.0	4.68	NDMA	94.3	4.67
NMEA	95.5	3.94	NMEA	104	5.18	NMEA	102	5.32
NDEA	90.0	3.29	NDEA	97.4	5.16	NDEA	97.3	4.63
NPIP	86.6	4.37	NPIP	92.8	4.88	NPIP	91.9	4.35
NPYR	111	6.80	NPYR	106	5.03	NPYR	100	4.55
NMOR	89.8	3.41	NMOR	95.6	4.43	NMOR	95.7	4.64
