# Supplementary material for: A New Automated Method and Sample Data Flow for Analysis of Volatile Nitrosamines in Human Urine
Source: Am J Analyt Chem. Author manuscript; Available in PMC 2017 Feb 1. (PMC4770837; doi:10.4236/ajac.2016.72014)
Supplement: 01 — Figure S1. Sample preparation work flow. Figure S2. Short-term stability of NDMA. Figure S3. Short-term stability of NMEA. Figure S4. Short-term stability of NDEA. Figure S5. Short-term stability of NPIP. Figure S6. Short-term stability of NPYR. Figure S7. Short-term stability of NMOR. Table S1. Matrix equivalency (All r2 values > 0.999). Table S2. Ruggedness. [file NIHMS758494-supplement-01.pdf]

Supplement

S1. Linearity

The standard curve shows linearity above 0.999 for a large dynamic range, from 0 to 400 ng/mL, with the lowest non-zero standard concentration being 0.1 ng/mL. The linear range of the analytical method extends from the LOD to the highest calibrator. A weighting factor of 1/x was used for all analytes. A full calibration curve is run with each analytical batch. The calibration curve is rejected if the R-squared value is less than 0.98 for the full dynamic range or the back calculated concentration of any standard is greater than ±10% of the nominal concentration.

S2. Matrix Equivalency

To verify matrix equivalency, 10 standard solutions were prepared in ACN (non-matrix). Another 10 standard solutions were prepared in urine (matrix), and carried through sample preparation as described in sample preparation section above. Each calibration curve was run in triplicate, and the average slopes for the two matrices were compared for each analyte. The differences in slope between the matrices for all analytes were below 5%, with NPYR having less than 0.5% difference (**Table S1**).

S3. Ruggedness

To test the ruggedness of both the sample preparation and the GC-QQQ methods, QC samples were prepared and run under different conditions in five parameters, tested separately. These parameters include the number of programmed loops in the pipette mixing step (where one loop is approximately 4 minutes), the volume of DCM added to each well, the volume of prepared sample injected onto the GC, the source temperature of the mass spectrometer, and the percentage of chemical ionization gas (ammonia) in the MS source. Samples were tested for all parameters at below, above, and normal operating conditions. For all parameters tested, less than a 20% difference in concentration was calculated for all analytes, with the majority well below 10% (**Table S2**).

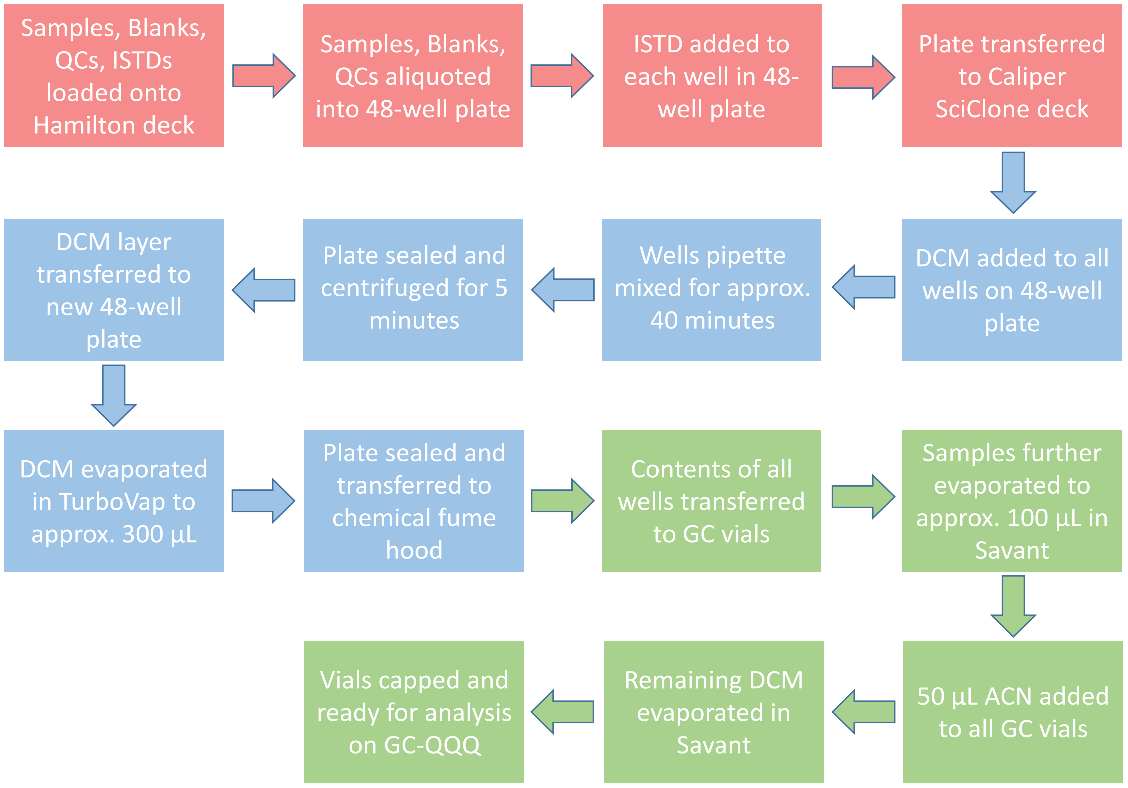

Figure S1. Sample preparation work flow.

Table S1. Matrix equivalency (All r^2 values > 0.999).

	Acetonitrile	Urine	% Diff
	Slope (n = 3)	Slope (n = 3)	
NDMA	0.903	0.928	2.79
NMEA	2.11	2.20	4.04
NDEA	1.14	1.17	3.00
NPIP	0.890	0.848	4.79
NPYR	1.29	1.29	0.478
NMOR	1.16	1.13	2.89

Table S2. Ruggedness.

	NDMA	NMEA	NDEA	NPIP	NPYR	NMOR
Pipette Mixing—8 loops	58.3 (+1.6%)	46.5 (+5.0%)	44.2 (+0.9%)	40.8 (+0.5%)	64.5 (+4.9%)	52.9 (+2.7%)
Pipette Mixing—10 loops*	57.4	44.3	43.8	40.6	61.5	51.5
Pipette Mixing—12 loops	58.9 (+2.6%)	46.8 (+5.6%)	47.0 (+7.3%)	43.6 (+7.4%)	61.9 (+0.7%)	52.7 (+2.3%)
DCM volume—2 mL	60.1 (−1.2%)	46.0 (−2.1%)	46.5 (−0.6%)	41.4 (−4.6%)	62.9 (−0.8%)	57.9 (+5.5%)
DCM volume—2.5 mL*	60.8	47.0	46.8	43.4	63.4	54.9
DCM volume—3 mL	59.8 (−1.6%)	38.4 (−18.3%)	42.0 (-10.3%)	35.7 (−17.7%)	56.3 (−11.2%)	44.0 (−19.9%)
Injection volume—4 µL	69.0 (−5.2%)	46.6 (+2.0%)	51.3 (+1.6%)	44.1 (+1.4%)	60.6 (−5.6%)	53.8 (−2.0%)
Injection volume—5 µL*	72.8	45.7	50.5	43.5	64.2	54.9
Injection volume—6 µL	76.8 (+5.5%)	46.4 (+1.5%)	50.8 (+0.6%)	44.1 (+1.4%)	63.6 (−0.9%)	53.1 (−3.3%)
Source temperature—225˚C	69.8 (−7.9%)	46.7 (+1.7%)	50.2 (−2.1%)	42.4 (−3.9%)	62.5 (−2.6%)	52.2 (−1.1%)
Source temperature—250˚C*	75.8	45.9	51.3	44.1	64.2	52.8
Source temperature—275˚C	85.0 (+12.1%)	47.9 (+4.4%)	51.9 (+1.2%)	46.6 (+5.7%)	63.8 (−0.6%)	53.8 (+1.9%)
CI gas—20%	62.5 (−3.1%)	40.7 (+3.3%)	43.5 (−1.4%)	37.5 (+1.4%)	58.2 (+2.3%)	48.4 (+1.7%)
CI gas—25%*	64.5	39.4	44.1	37.0	56.9	47.6
CI gas—30%	74.6 (+15.7%)	40.7 (+3.3%)	43.2 (−2.0%)	35.8 (−3.2%)	57.5 (+1.0%)	45.9 (−3.6%)

All values in pg/mL. *Denotes current method setting.

S4. Stability

To ensure the stability of all analytes, two freshly made QC pools (50 pg/mL, 200 pg/mL) were analyzed daily over a 30 day period, with day zero representing the day the pools were made. No significant decrease in concentration is observed for any analyte at either concentration over the duration of the study (**Figures S2-S7**).

S5. QC Characterization

The two QC pools used for this method, 50 pg/mL and 200 pg/mL, were characterized using 20 replicates for each pool over 20 days. QC results were subsequently used to verify methodological precision for each analytical run according to modified Westgard QC rules [30].

S6. Proficiency Testing

Proficiency testing was conducted by analyzing a series of 5 "blinded" VNA-spiked urine samples every 6 months. PT testing samples were blind-coded by a statistician or QC officer. If blind-analyzed concentrations fell within 20% of known values for all analyte, and the overall passing rate is 80% or greater, then the PT was passed. No sample analysis is permitted until PT is passed.

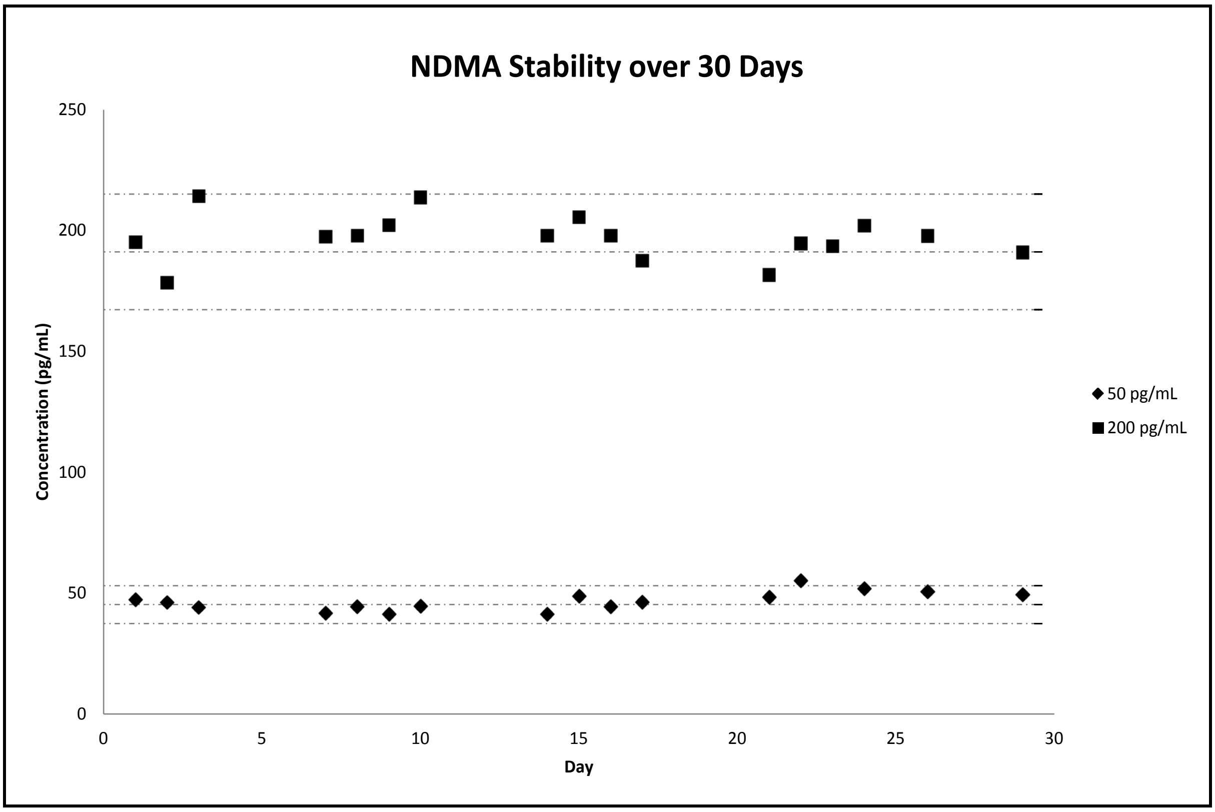

Figure S2. Short-term stability of NDMA.

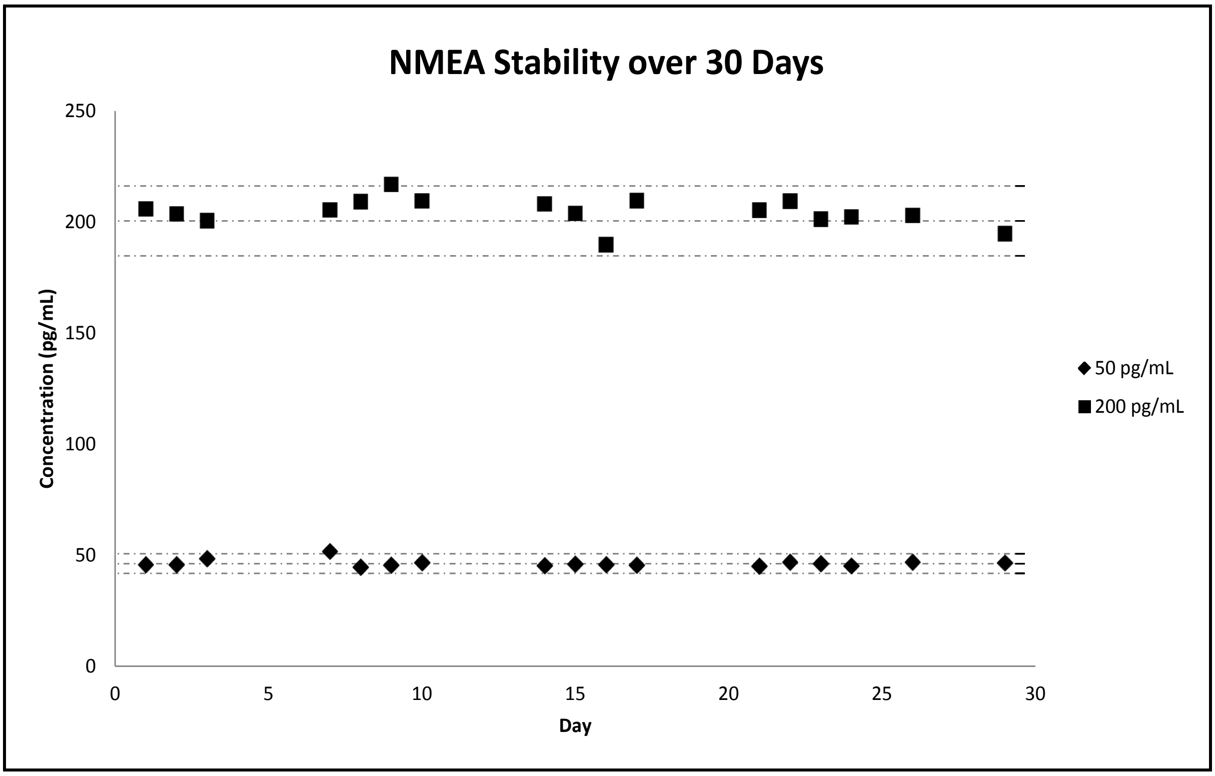

Figure S3. Short-term stability of NMEA.

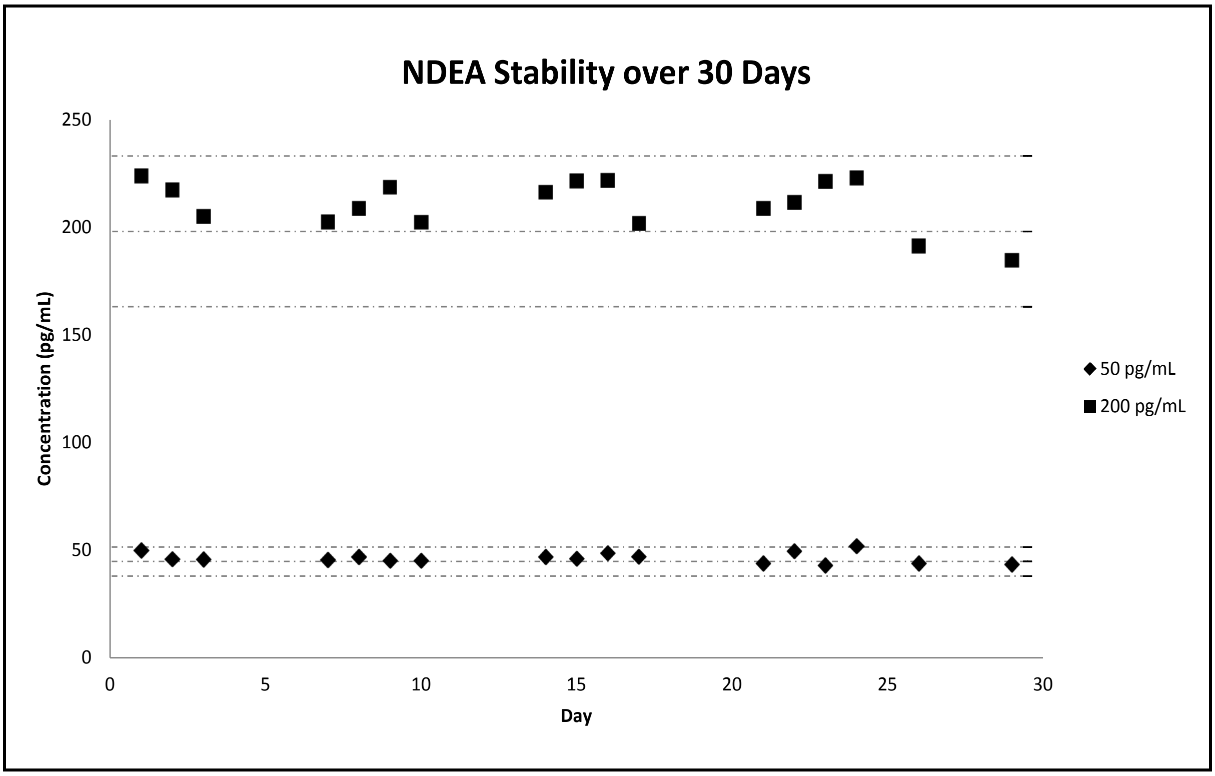

Figure S4. Short-term stability of NDEA.

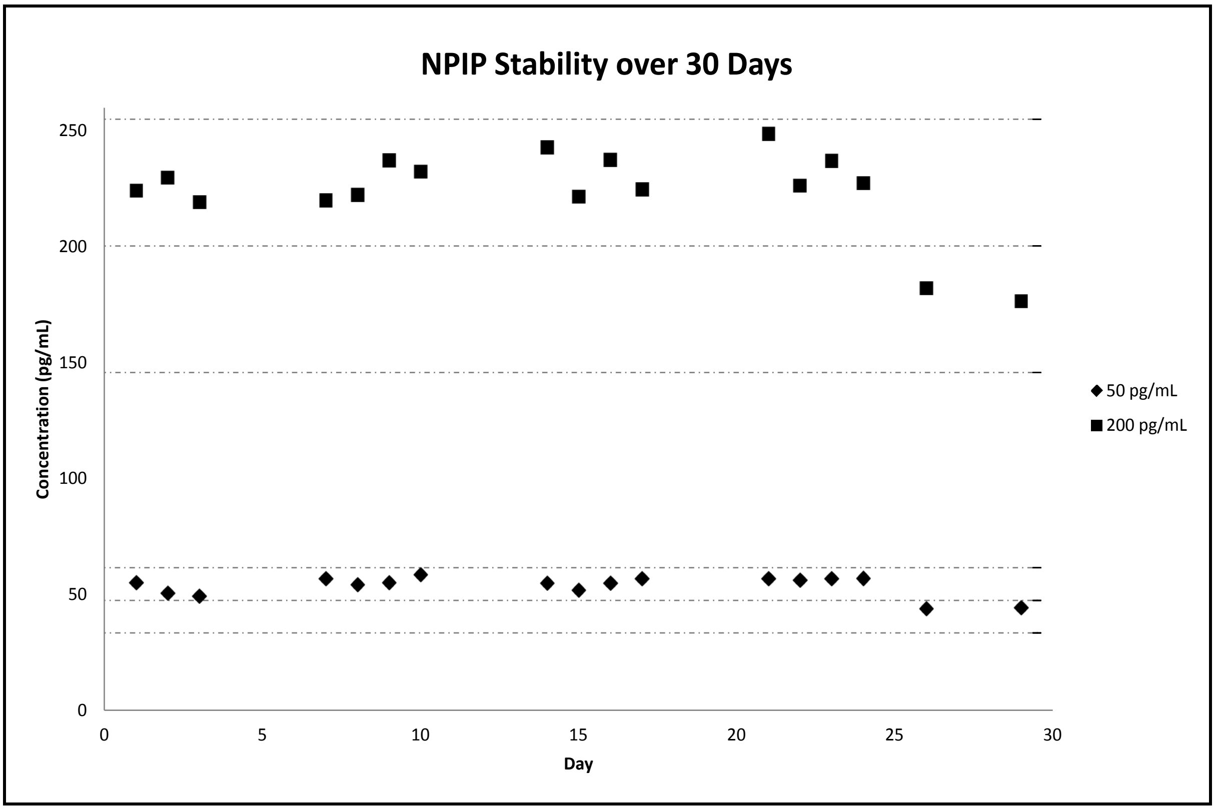

Figure S5. Short-term stability of NPIP.

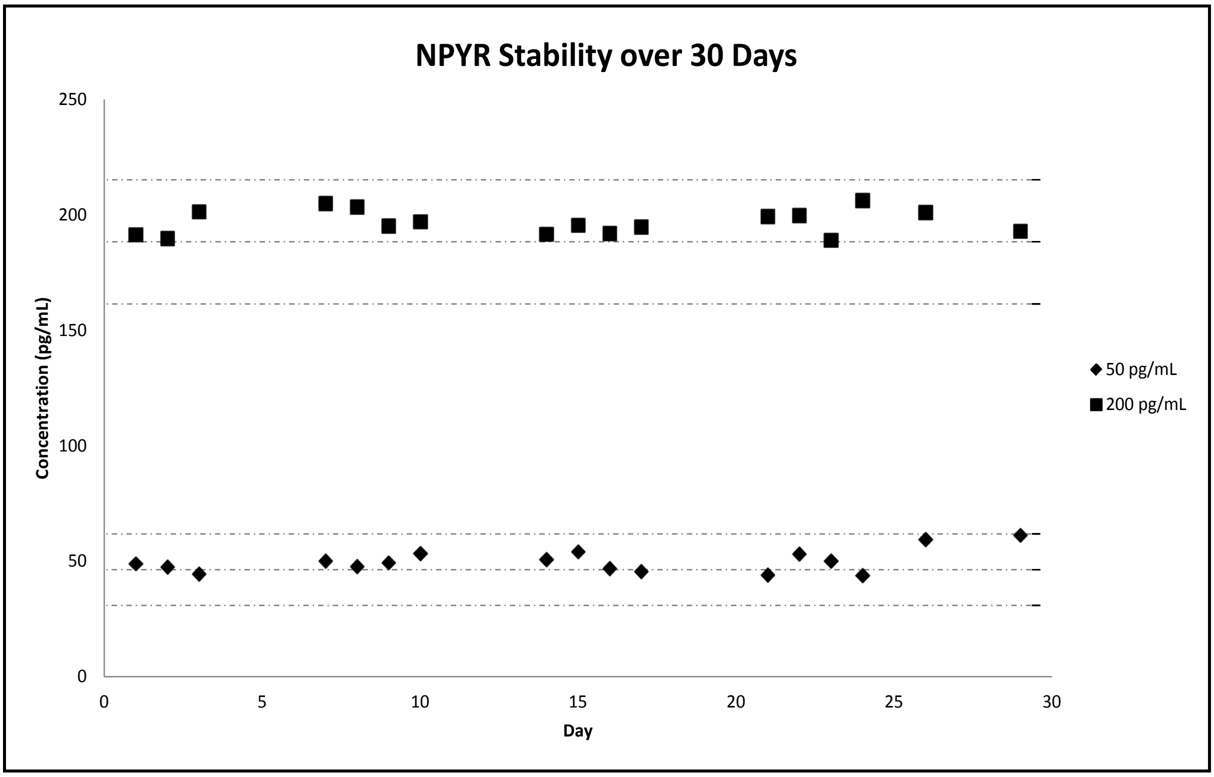

Figure S6. Short-term stability of NPYR.

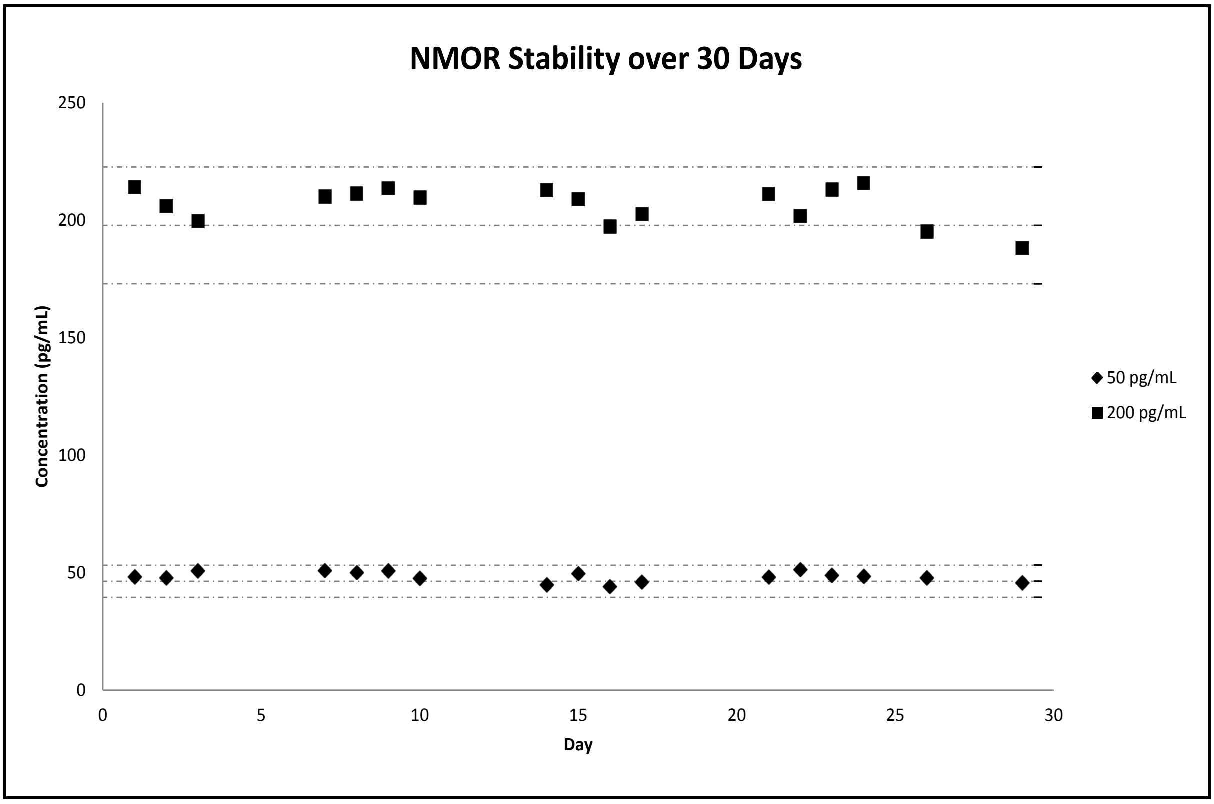

Figure S7. Short-term stability of NMOR.
